# Tetra­ethyl­ammonium dicyanido(5,10,15,20-tetra­phenyl­porphyrinato)ferrate(III) di­chloro­methane monosolvate

**DOI:** 10.1107/S1600536813019119

**Published:** 2013-07-17

**Authors:** Nazira Kassenova, Oleksandr Hietsoi, Rakhmetulla Yerkassov, Michael Shatruk

**Affiliations:** a Department of Chemistry, L.N. Gumilyov Eurasian National University, 5 Munaitpasov Str, 010008 Astana, Kazakhstan; b Department of Chemistry and Biochemistry, Florida State University, 95 Chieftan Way, Tallahassee, FL 32306-4390, USA

## Abstract

The title compound, (C_8_H_20_N)[Fe(C_44_H_28_N_4_)(CN)_2_]·CH_2_Cl_2_ or (Et_4_N)[Fe(TPP)(CN)_2_], was recrystallized from di­chloro­methane–diethyl ether. The compound crystallizes with the two unique halves of the Fe^III^ porphyrinato complex, one tetra­ethyl­ammonium cation and one inter­stitial di­chloro­methane mol­ecule within the asymmetric unit. Both anionic Fe^III^ complexes exhibit inversion symmetry. Both the cation and the solvent mol­ecules show positional disorder. The cation is disordered over two sets of sites with an occupancy ratio of 0.710 (3):0.290 (3); the solvent mol­ecule is disordered over three positions with a 0.584 (6):0.208 (3):0.202 (5) ratio. The crystal packing features columns of [Fe(TPP)(CN)_2_]^−^ anions that propagate along [001]. The columns further pack into layers that are parallel to (011) and also include the Et_4_N^+^ cations. The inter­stitial CH_2_Cl_2_ mol­ecules appear in the inter­layer space. This complex may serve as a useful precursor for the assembly of multinuclear and extended CN-bridged complexes for the design of single-mol­ecule and single-chain magnets, respectively.

## Related literature

For transition metal ions bridged by cyanide, see: Corsi *et al.* (1999 ▶); Dunbar & Heintz (1997 ▶); Scott *et al.* (1994 ▶); Schelter *et al.* (2004 ▶, 2007 ▶); Shatruk *et al.* (2009 ▶). For similar porphyrin compounds, see: Li *et al.* (2009 ▶); Scheidt *et al.* (1980 ▶).
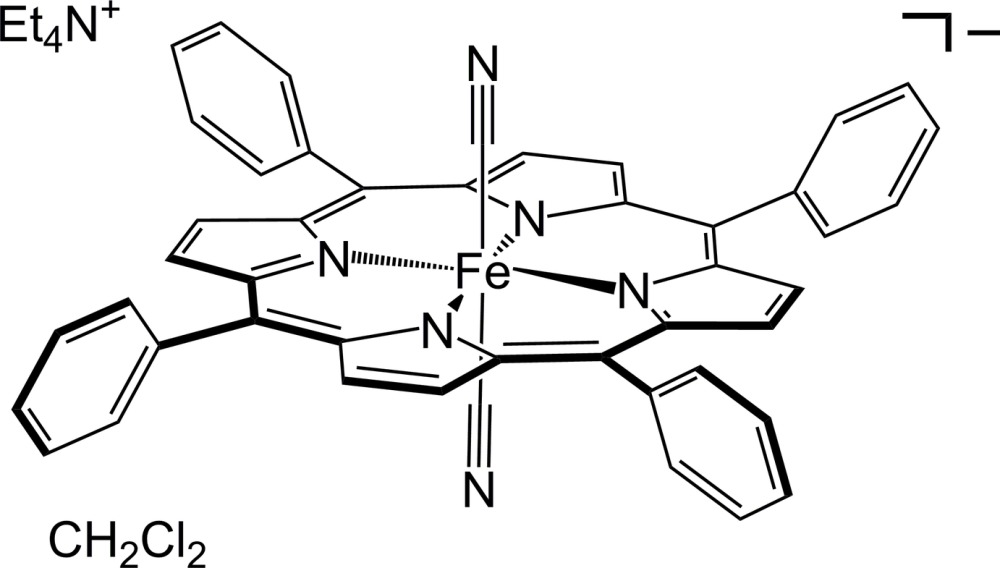


## Experimental

### 

#### Crystal data

(C_8_H_20_N)[Fe(C_44_H_28_N_4_)(CN)_2_]·CH_2_Cl_2_
*M* *_r_* = 935.77
Triclinic, 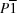

*a* = 11.0069 (8) Å
*b* = 15.0344 (11) Å
*c* = 15.4350 (11) Å
α = 80.075 (1)°
β = 77.527 (1)°
γ = 83.077 (1)°
*V* = 2447.4 (3) Å^3^
*Z* = 2
Mo *K*α radiation
μ = 0.46 mm^−1^
*T* = 173 K
0.22 × 0.15 × 0.09 mm

#### Data collection

Bruker APEXII CCD area-detector diffractometer
Absorption correction: numerical (*SADABS*; Bruker, 2003 ▶) *T*_min_ = 0.905, *T*_max_ = 0.960
19913 measured reflections
9891 independent reflections
7117 reflections with *I* > 2σ(*I*)
*R*_int_ = 0.034

#### Refinement

*R*[*F* ^2^ > 2σ(*F* ^2^)] = 0.050
*wR*(*F* ^2^) = 0.129
*S* = 1.12
9891 reflections
682 parameters
37 restraints
H atoms treated by a mixture of independent and constrained refinement
Δρ_max_ = 0.40 e Å^−3^
Δρ_min_ = −0.43 e Å^−3^

### 

Data collection: *SMART* (Bruker, 2003 ▶); cell refinement: *SAINT* (Bruker, 2003 ▶); data reduction: *SAINT*; program(s) used to solve structure: *SHELXS97* (Sheldrick, 2008 ▶); program(s) used to refine structure: *SHELXL97* (Sheldrick, 2008 ▶); molecular graphics: *X-SEED* (Barbour, 2001 ▶); software used to prepare material for publication: *SHELXL97*.

## Supplementary Material

Crystal structure: contains datablock(s) I, global. DOI: 10.1107/S1600536813019119/pj2003sup1.cif

Structure factors: contains datablock(s) I. DOI: 10.1107/S1600536813019119/pj2003Isup2.hkl

Additional supplementary materials:  crystallographic information; 3D view; checkCIF report

## Figures and Tables

**Fig. 1.**
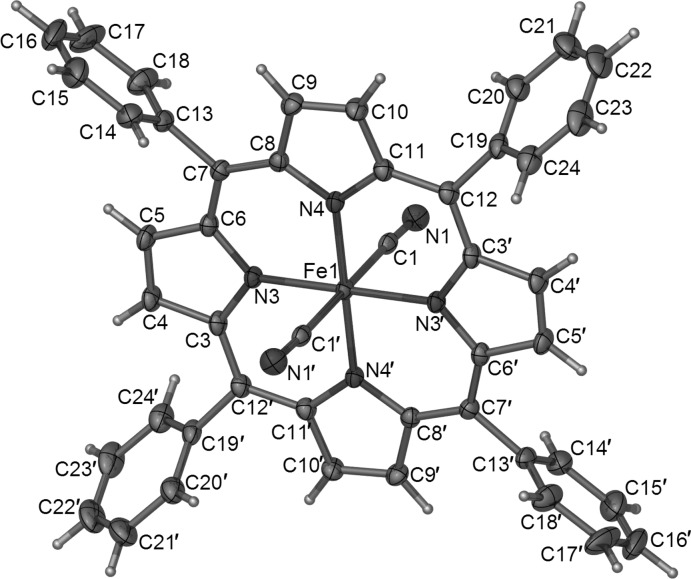
Approximately octahedral coordination about one FeIII. Displacement ellipsoids are drawn at the 50% probability level.

## supplementary crystallographic information

### Comment

Mononuclear metal complexes with terminal cyanide ligands have been shown to be useful building blocks for the construction of coordination oligomers and polymers based on the CN-bridged transition metal ions.(Dunbar & Heintz, 1997) A particular interest in these materials stems from the possibility of a judicious design and preparation of magnetically bistable materials, such as single-molecule and single-chain magnets (SMMs and SCMs). (Shatruk *et al.*, 2009) A prerequisite for the existence of SMM and SCM properties is a significant magnetic anisotropy and high ground-state spin value of the transition metal ion. An effective approach uses a combination of two monometallic building blocks to satisfy both these criteria in the final structure.

A number of recent approaches have considered the mononuclear CN-terminated complexes as metalloligands that can be combined with solvated or partially ligated metal ions to create specific molecular shapes in a predictable (modular) manner.(Schelter *et al.*, 2004 and Schelter *et al.*, 2007) With the goal to prepare such metalloligand that would incorporate a magnetically anisotropic metal center, we turned to iron(III) tetraphenylporphyrinato anion, [Fe(TPP)]^-^ (S = 1/2). It was reported that a reaction between [Fe(TPP)Cl] and KCN leads to the desired salt, K[Fe(TPP)(CN)_2_], and the crystal structure of this salt was established, (Scheidt *et al.*,1980) as well as the structure of its close analogue, in which the K^+^ ion was ligated by 18-crown-6 macrocycle.(Li *et al.*, 2009) Nevertheless, reports on oligomeric or polymeric CN-bridged structures obtained with the [Fe(TPP)_2_(CN)_2_]^-^ building block are very scarce.(Scott *et al.*, 1994 and Corsi *et al.*, 1999) Therefore, we set out to obtain a convenient, readily soluble precursor that could be used for the preparation of such structures.

A metathesis reaction between K[Fe(TPP)(CN)_2_] and (Et_4_N)Cl led to the isolation of dark-violet solid, (Et_4_N)[Fe(TPP)(CN)_2_] that could be readily recrystallized from CH_2_Cl_2_/Et_2_O. The compound is soluble in a variety of organic solvents, including dichloromethane, chloroform, acetonitrile, acetone, and *N*,*N*'-dimethylformamide. The results of our current efforts to use this precursor in the preparation of CN-bridged multinuclear assemblies will be reported in due course.

### Experimental

*Caution: Potassium cyanide (KCN) used in this preparation is extremely poisonous. It should be used in small amounts and handled with great care!*

[Fe(TPP)Cl] (200 mg, 0.284 mmol) was dissolved in 50 mL of methanol. To this solution was added an excess of KCN (185 mg, 2.84 mmol), and the reaction mixture was stirred under reflux for 12 h. After cooling down to room temperature, the obtained dark-violet solid was recovered by filtration and washed thoroughly with copious amount of water to remove the remaining KCN. The filter cake was redissolved in 200 mL of acetonitrile and filtered. The filtrate was evaporated to dryness. The dark-violet solid residue was redissolved in 50 mL of dichloromethane and layered with an equal volume of diethyl ether in a Schlenk tube. Within a few hours, needle-like dark-violet crystals of (Et_4_N)[Fe(TPP)(CN)_2_] appeared in the the tube. The crystals were harvested once the diffusion of Et_2_O into the solution of the complex was complete.

### Refinement

The tetraethylammonium cation was disordered over two positions around the central N atom, which were refined under the constraint that the total occupancy of both position is equal to 1. The interstitial dichloromethane molecule was disordered over three positions, the total occupancy of which also was set equal to 1. (An attempt to refine the CH_2_Cl_2_ molecule with only two disorder components consisently led to the appearance of significant peaks in the difference Fourier electron density maps.) The isotropic atomic displacement parameters (ADPs) of all C atoms from the three disorder components of the CH_2_Cl_2_ molecule were set equal, in order to minimize the correlation with the site occupancy factors (SOFs) and taking into account that these atoms were located nearby one another. The Cl atoms of the CH_2_Cl_2_ molecule were refined anisotropically, since the isotropic refinement consistently led to the appearance of significant residual electron density peaks in the vicinity of these atoms. To minimize the correlation between the ADPs and SOFs, the ADPs of the disordered Cl atoms that appeared closer than 1.2 Å to each other were restricted to be similar, using the SIMU instruction in *SHELXL*.

### Crystal data

**Table:**

(C_8_H_20_N)[Fe(C_44_H_28_N_4_)(CN)_2_]·CH_2_Cl_2_	*V* = 2447.4 (3) Å^3^
*M**_r_* = 935.77	*Z* = 2
Triclinic, *P*1	*F*(000) = 978
Hall symbol: -P 1	*D*_x_ = 1.270 Mg m^−^^3^
*a* = 11.0069 (8) Å	Mo *K*α radiation, λ = 0.71073 Å
*b* = 15.0344 (11) Å	Cell parameters from 374 reflections
*c* = 15.4350 (11) Å	µ = 0.46 mm^−^^1^
α = 80.075 (1)°	*T* = 173 K
β = 77.527 (1)°	Block, violet
γ = 83.077 (1)°	0.22 × 0.15 × 0.09 mm

### Data collection

**Table:**

Bruker APEXII CCD area-detector diffractometer	9891 independent reflections
Radiation source: fine-focus sealed tube	7117 reflections with *I* > 2σ(*I*)
Graphite monochromator	*R*_int_ = 0.034
profile data from ω scans	θ_max_ = 26.4°, θ_min_ = 1.8°
Absorption correction: numerical (*SADABS*; Bruker, 2003)	*h* = −13→13
*T*_min_ = 0.905, *T*_max_ = 0.960	*k* = −18→18
19913 measured reflections	*l* = −19→19

### Refinement

**Table:**

Refinement on *F*^2^	Primary atom site location: structure-invariant direct methods
Least-squares matrix: full	Secondary atom site location: difference Fourier map
*R*[*F*^2^ > 2σ(*F*^2^)] = 0.050	Hydrogen site location: inferred from neighbouring sites
*wR*(*F*^2^) = 0.129	H atoms treated by a mixture of independent and constrained refinement
*S* = 1.12	*w* = 1/[σ^2^(*F*_o_^2^) + (0.0577*P*)^2^] where *P* = (*F*_o_^2^ + 2*F*_c_^2^)/3
9891 reflections	(Δ/σ)_max_ = 0.011
682 parameters	Δρ_max_ = 0.40 e Å^−^^3^
37 restraints	Δρ_min_ = −0.43 e Å^−^^3^

### Special details

**Table:**

Geometry. All e.s.d.'s (except the e.s.d. in the dihedral angle between two l.s. planes) are estimated using the full covariance matrix. The cell e.s.d.'s are taken into account individually in the estimation of e.s.d.'s in distances, angles and torsion angles; correlations between e.s.d.'s in cell parameters are only used when they are defined by crystal symmetry. An approximate (isotropic) treatment of cell e.s.d.'s is used for estimating e.s.d.'s involving l.s. planes.
Refinement. Refinement of *F*^2^ against ALL reflections. The weighted *R*-factor *wR* and goodness of fit *S* are based on *F*^2^, conventional *R*-factors *R* are based on *F*, with *F* set to zero for negative *F*^2^. The threshold expression of *F*^2^ > σ(*F*^2^) is used only for calculating *R*-factors(gt) *etc*. and is not relevant to the choice of reflections for refinement. *R*-factors based on *F*^2^ are statistically about twice as large as those based on *F*, and *R*- factors based on ALL data will be even larger.

### Fractional atomic coordinates and isotropic or equivalent isotropic displacement parameters (Å^2^)

**Table:**

	*x*	*y*	*z*	*U*_iso_*/*U*_eq_	Occ. (<1)
Fe1	0.0000	0.5000	0.0000	0.01869 (13)	
Fe2	0.5000	0.0000	0.5000	0.01952 (13)	
N1	−0.1628 (2)	0.42371 (15)	0.18511 (15)	0.0333 (5)	
N2	0.2623 (2)	−0.04837 (16)	0.44363 (15)	0.0379 (6)	
N3	0.14203 (17)	0.41953 (12)	0.03907 (12)	0.0205 (4)	
N4	0.04346 (17)	0.59835 (12)	0.05767 (12)	0.0197 (4)	
N5	0.46273 (17)	0.12961 (13)	0.44784 (12)	0.0215 (4)	
N6	0.60197 (17)	−0.02392 (13)	0.38148 (12)	0.0218 (4)	
N7	0.00444 (19)	0.17939 (14)	0.34375 (14)	0.0297 (5)	
C1	−0.1057 (2)	0.45270 (16)	0.11609 (17)	0.0220 (5)	
C2	0.3502 (2)	−0.03109 (16)	0.46476 (15)	0.0242 (5)	
C3	0.1698 (2)	0.32906 (16)	0.03127 (15)	0.0220 (5)	
C4	0.2723 (2)	0.29234 (17)	0.07419 (16)	0.0267 (6)	
H4	0.3093	0.2317	0.0778	0.032*	
C5	0.3062 (2)	0.36000 (16)	0.10847 (17)	0.0279 (6)	
H5	0.3723	0.3562	0.1400	0.033*	
C6	0.2240 (2)	0.43871 (16)	0.08838 (15)	0.0216 (5)	
C7	0.2201 (2)	0.51980 (16)	0.12243 (15)	0.0219 (5)	
C8	0.1332 (2)	0.59287 (15)	0.10891 (15)	0.0207 (5)	
C9	0.1306 (2)	0.67796 (16)	0.14082 (16)	0.0254 (6)	
H9	0.1812	0.6913	0.1788	0.030*	
C10	0.0428 (2)	0.73499 (16)	0.10639 (16)	0.0258 (6)	
H10	0.0207	0.7964	0.1150	0.031*	
C11	−0.0111 (2)	0.68592 (15)	0.05439 (15)	0.0223 (5)	
C12	−0.1068 (2)	0.72216 (16)	0.00936 (16)	0.0229 (5)	
C13	0.3131 (2)	0.52463 (16)	0.17977 (17)	0.0257 (6)	
C14	0.2745 (3)	0.52788 (18)	0.27039 (18)	0.0372 (7)	
H14	0.1879	0.5300	0.2964	0.045*	
C15	0.3606 (3)	0.5280 (2)	0.3240 (2)	0.0526 (9)	
H15	0.3327	0.5308	0.3862	0.063*	
C16	0.4861 (4)	0.5242 (2)	0.2874 (3)	0.0601 (10)	
H16	0.5448	0.5234	0.3246	0.072*	
C17	0.5266 (3)	0.5217 (2)	0.1978 (3)	0.0596 (10)	
H17	0.6135	0.5194	0.1726	0.072*	
C18	0.4402 (2)	0.52258 (19)	0.1431 (2)	0.0414 (7)	
H18	0.4686	0.5218	0.0805	0.050*	
C19	−0.1397 (2)	0.82238 (17)	−0.00045 (16)	0.0271 (6)	
C20	−0.0519 (3)	0.87988 (17)	−0.05242 (18)	0.0355 (7)	
H20	0.0295	0.8552	−0.0765	0.043*	
C21	−0.0822 (3)	0.97271 (19)	−0.0692 (2)	0.0467 (8)	
H21	−0.0215	1.0110	−0.1045	0.056*	
C22	−0.1998 (3)	1.0094 (2)	−0.0350 (2)	0.0501 (9)	
H22	−0.2210	1.0728	−0.0476	0.060*	
C23	−0.2868 (3)	0.9538 (2)	0.0175 (2)	0.0457 (8)	
H23	−0.3678	0.9792	0.0418	0.055*	
C24	−0.2568 (3)	0.86053 (18)	0.03540 (18)	0.0357 (7)	
H24	−0.3172	0.8229	0.0724	0.043*	
C25	0.3902 (2)	0.19649 (16)	0.49195 (16)	0.0234 (5)	
C26	0.3856 (2)	0.27988 (17)	0.43057 (17)	0.0310 (6)	
H26	0.3441	0.3363	0.4445	0.037*	
C27	0.4508 (2)	0.26329 (16)	0.35002 (17)	0.0291 (6)	
H27	0.4624	0.3056	0.2962	0.035*	
C28	0.5000 (2)	0.16996 (16)	0.36010 (16)	0.0233 (5)	
C29	0.5730 (2)	0.12724 (16)	0.29087 (15)	0.0234 (5)	
C30	0.6228 (2)	0.03713 (16)	0.30303 (15)	0.0227 (5)	
C31	0.7066 (2)	−0.00506 (17)	0.23383 (16)	0.0287 (6)	
H31	0.7364	0.0228	0.1741	0.034*	
C32	0.7351 (2)	−0.09116 (17)	0.26913 (16)	0.0281 (6)	
H32	0.7891	−0.1354	0.2391	0.034*	
C33	0.6684 (2)	−0.10386 (16)	0.36095 (15)	0.0229 (5)	
C34	0.6721 (2)	−0.18564 (16)	0.41893 (16)	0.0251 (6)	
C35	0.5930 (2)	0.17737 (16)	0.19703 (16)	0.0246 (5)	
C36	0.5446 (2)	0.14431 (17)	0.13316 (16)	0.0265 (6)	
H36	0.5002	0.0915	0.1503	0.032*	
C37	0.5608 (2)	0.18797 (17)	0.04515 (16)	0.0290 (6)	
H37	0.5285	0.1646	0.0021	0.035*	
C38	0.6235 (2)	0.26498 (19)	0.02011 (18)	0.0345 (6)	
H38	0.6333	0.2954	−0.0399	0.041*	
C39	0.6723 (2)	0.29803 (19)	0.08211 (18)	0.0356 (7)	
H39	0.7166	0.3508	0.0643	0.043*	
C40	0.6571 (2)	0.25502 (17)	0.16997 (17)	0.0302 (6)	
H40	0.6907	0.2786	0.2122	0.036*	
C41	0.7494 (2)	−0.26561 (17)	0.38376 (16)	0.0291 (6)	
C42	0.7143 (3)	−0.3075 (2)	0.3214 (2)	0.0585 (10)	
H42	0.6396	−0.2854	0.3006	0.070*	
C43	0.7865 (3)	−0.3816 (2)	0.2883 (3)	0.0655 (11)	
H43	0.7602	−0.4099	0.2458	0.079*	
C44	0.8940 (3)	−0.4134 (2)	0.3167 (2)	0.0540 (9)	
H44	0.9434	−0.4640	0.2943	0.065*	
C45	0.9299 (4)	−0.3726 (3)	0.3767 (3)	0.0819 (13)	
H45	1.0050	−0.3949	0.3970	0.098*	
C46	0.8588 (3)	−0.2983 (2)	0.4095 (2)	0.0639 (11)	
H46	0.8873	−0.2698	0.4509	0.077*	
C47A	0.0168 (4)	0.2646 (3)	0.2738 (3)	0.0416 (11)	0.710 (3)
H47A	−0.0657	0.2864	0.2587	0.050*	0.710 (3)
H47B	0.0747	0.2502	0.2184	0.050*	0.710 (3)
C47B	0.0593 (8)	0.2357 (6)	0.3891 (6)	0.034 (2)	0.290 (3)
H47C	0.1433	0.2095	0.3982	0.041*	0.290 (3)
H47D	0.0057	0.2448	0.4478	0.041*	0.290 (3)
C48A	0.0676 (3)	0.3408 (2)	0.3107 (3)	0.0724 (12)	0.710 (3)
H48A	0.0074	0.3580	0.3631	0.109*	0.710 (3)
H48B	0.0796	0.3939	0.2639	0.109*	0.710 (3)
H48C	0.1476	0.3182	0.3280	0.109*	0.710 (3)
C48B	0.0676 (3)	0.3408 (2)	0.3107 (3)	0.0724 (12)	0.29
H48D	0.0140	0.3892	0.3388	0.109*	0.290 (3)
H48E	0.0394	0.3329	0.2567	0.109*	0.290 (3)
H48F	0.1541	0.3572	0.2942	0.109*	0.290 (3)
C49A	−0.0808 (4)	0.1996 (3)	0.4304 (3)	0.0385 (10)	0.710 (3)
H49A	−0.0400	0.2393	0.4584	0.046*	0.710 (3)
H49B	−0.0933	0.1423	0.4724	0.046*	0.710 (3)
C49B	−0.1269 (8)	0.2211 (7)	0.3257 (6)	0.036 (2)	0.290 (3)
H49C	−0.1668	0.1766	0.3031	0.043*	0.290 (3)
H49D	−0.1174	0.2757	0.2796	0.043*	0.290 (3)
C50A	−0.2114 (3)	0.2472 (2)	0.4158 (2)	0.0580 (9)	0.710 (3)
H50A	−0.2003	0.3066	0.3788	0.087*	0.710 (3)
H50B	−0.2651	0.2549	0.4740	0.087*	0.710 (3)
H50C	−0.2505	0.2096	0.3854	0.087*	0.710 (3)
C50B	−0.2114 (3)	0.2472 (2)	0.4158 (2)	0.0580 (9)	0.29
H50D	−0.2720	0.2982	0.4018	0.087*	0.290 (3)
H50E	−0.1588	0.2645	0.4528	0.087*	0.290 (3)
H50F	−0.2560	0.1950	0.4486	0.087*	0.290 (3)
C51A	−0.0429 (4)	0.1098 (3)	0.3038 (3)	0.0461 (12)	0.710 (3)
H51A	0.0114	0.1037	0.2446	0.055*	0.710 (3)
H51B	−0.1280	0.1316	0.2936	0.055*	0.710 (3)
C51B	−0.0146 (8)	0.0877 (6)	0.4082 (6)	0.028 (2)	0.290 (3)
H51C	0.0631	0.0665	0.4309	0.034*	0.290 (3)
H51D	−0.0821	0.0978	0.4603	0.034*	0.290 (3)
C52A	−0.0476 (3)	0.0148 (2)	0.3627 (2)	0.0537 (9)	0.710 (3)
H52A	0.0367	−0.0084	0.3713	0.081*	0.710 (3)
H52B	−0.0802	−0.0268	0.3327	0.081*	0.710 (3)
H52C	−0.1022	0.0199	0.4212	0.081*	0.710 (3)
C52B	−0.0476 (3)	0.0148 (2)	0.3627 (2)	0.0537 (9)	0.29
H52D	−0.1333	0.0287	0.3529	0.081*	0.290 (3)
H52E	−0.0408	−0.0440	0.4009	0.081*	0.290 (3)
H52F	0.0100	0.0128	0.3048	0.081*	0.290 (3)
C53A	0.1330 (3)	0.1462 (3)	0.3667 (3)	0.0353 (10)	0.710 (3)
H53A	0.1256	0.0918	0.4132	0.042*	0.710 (3)
H53B	0.1643	0.1940	0.3907	0.042*	0.710 (3)
C53B	0.0757 (5)	0.1613 (6)	0.2532 (6)	0.037 (2)	0.290 (3)
H53C	0.0788	0.2171	0.2083	0.044*	0.290 (3)
H53D	0.0399	0.1141	0.2314	0.044*	0.290 (3)
C54A	0.2278 (3)	0.1224 (3)	0.2790 (3)	0.0806 (14)	0.710 (3)
H54A	0.1887	0.0851	0.2484	0.121*	0.710 (3)
H54B	0.3035	0.0889	0.2956	0.121*	0.710 (3)
H54C	0.2498	0.1786	0.2387	0.121*	0.710 (3)
C54B	0.2278 (3)	0.1224 (3)	0.2790 (3)	0.0806 (14)	0.29
C1A	0.3425 (7)	0.8040 (7)	0.3096 (5)	0.074 (2)*	0.584 (6)
H1A1	0.3902	0.7452	0.3245	0.089*	0.584 (6)
H1A2	0.3516	0.8434	0.3527	0.089*	0.584 (6)
Cl1A	0.1862 (3)	0.7849 (3)	0.3279 (3)	0.1086 (13)	0.584 (6)
Cl2A	0.4121 (5)	0.8529 (5)	0.2030 (3)	0.184 (3)	0.584 (6)
C1B	0.3142 (15)	0.8672 (12)	0.2567 (16)	0.074 (2)*	0.202 (5)
H1B1	0.3383	0.8787	0.3117	0.089*	0.202 (5)
H1B2	0.3156	0.9246	0.2141	0.089*	0.202 (5)
Cl1B	0.1710 (10)	0.8330 (9)	0.2823 (10)	0.127 (4)	0.202 (5)
Cl2B	0.4202 (7)	0.7859 (7)	0.2089 (6)	0.088 (3)	0.202 (5)
C1C	0.409 (2)	0.8496 (11)	0.2754 (14)	0.074 (2)*	0.208 (3)
H1C1	0.5006	0.8431	0.2727	0.089*	0.208 (3)
H1C2	0.3695	0.8831	0.3255	0.089*	0.208 (3)
Cl1C	0.3561 (15)	0.7451 (6)	0.2959 (10)	0.208 (8)	0.208 (3)
Cl2C	0.3785 (13)	0.9107 (7)	0.1765 (6)	0.142 (4)	0.208 (3)
H54D	0.2700 (17)	0.079 (2)	0.2397 (19)	0.213*	0.290 (3)
H54E	0.2180 (7)	0.093 (3)	0.3417 (8)	0.213*	0.290 (3)
H54F	0.2780 (15)	0.1741 (6)	0.269 (3)	0.213*	0.290 (3)

### Atomic displacement parameters (Å^2^)

**Table:**

	*U*^11^	*U*^22^	*U*^33^	*U*^12^	*U*^13^	*U*^23^
Fe1	0.0177 (3)	0.0193 (3)	0.0194 (3)	0.00194 (19)	−0.0052 (2)	−0.0044 (2)
Fe2	0.0203 (3)	0.0192 (3)	0.0185 (3)	−0.0006 (2)	−0.0025 (2)	−0.0039 (2)
N1	0.0309 (13)	0.0365 (13)	0.0309 (13)	−0.0030 (10)	−0.0021 (11)	−0.0060 (11)
N2	0.0343 (14)	0.0445 (15)	0.0361 (14)	−0.0105 (11)	−0.0102 (11)	0.0000 (11)
N3	0.0194 (10)	0.0203 (10)	0.0219 (11)	0.0028 (8)	−0.0063 (8)	−0.0043 (8)
N4	0.0198 (10)	0.0205 (10)	0.0191 (10)	0.0013 (8)	−0.0053 (8)	−0.0039 (8)
N5	0.0224 (11)	0.0208 (11)	0.0203 (11)	−0.0019 (8)	−0.0014 (9)	−0.0041 (8)
N6	0.0229 (11)	0.0207 (11)	0.0205 (11)	0.0010 (9)	−0.0033 (9)	−0.0033 (8)
N7	0.0271 (12)	0.0315 (12)	0.0293 (12)	−0.0027 (10)	−0.0089 (10)	0.0034 (10)
C1	0.0200 (13)	0.0213 (13)	0.0272 (14)	0.0036 (10)	−0.0099 (11)	−0.0076 (11)
C2	0.0278 (14)	0.0239 (13)	0.0177 (13)	−0.0010 (11)	−0.0011 (11)	0.0007 (10)
C3	0.0192 (12)	0.0235 (13)	0.0217 (13)	0.0068 (10)	−0.0045 (10)	−0.0043 (10)
C4	0.0245 (14)	0.0253 (13)	0.0303 (14)	0.0087 (11)	−0.0091 (11)	−0.0071 (11)
C5	0.0225 (13)	0.0310 (15)	0.0320 (15)	0.0063 (11)	−0.0125 (11)	−0.0071 (12)
C6	0.0180 (12)	0.0242 (13)	0.0214 (13)	0.0031 (10)	−0.0043 (10)	−0.0028 (10)
C7	0.0185 (12)	0.0242 (13)	0.0233 (13)	−0.0010 (10)	−0.0055 (10)	−0.0035 (10)
C8	0.0183 (12)	0.0230 (13)	0.0199 (12)	−0.0020 (10)	−0.0032 (10)	−0.0015 (10)
C9	0.0282 (14)	0.0284 (14)	0.0224 (13)	−0.0022 (11)	−0.0081 (11)	−0.0082 (11)
C10	0.0295 (14)	0.0216 (13)	0.0280 (14)	0.0021 (11)	−0.0080 (11)	−0.0087 (11)
C11	0.0232 (13)	0.0213 (13)	0.0222 (13)	0.0014 (10)	−0.0047 (10)	−0.0049 (10)
C12	0.0223 (13)	0.0218 (13)	0.0238 (13)	0.0038 (10)	−0.0042 (10)	−0.0060 (10)
C13	0.0227 (13)	0.0217 (13)	0.0352 (15)	0.0023 (10)	−0.0122 (11)	−0.0061 (11)
C14	0.0432 (17)	0.0341 (16)	0.0395 (17)	−0.0068 (13)	−0.0173 (14)	−0.0060 (13)
C15	0.072 (2)	0.048 (2)	0.048 (2)	−0.0080 (18)	−0.0355 (18)	−0.0036 (16)
C16	0.069 (3)	0.047 (2)	0.083 (3)	0.0031 (18)	−0.059 (2)	−0.0119 (19)
C17	0.0314 (17)	0.056 (2)	0.106 (3)	0.0034 (15)	−0.035 (2)	−0.030 (2)
C18	0.0271 (15)	0.0437 (17)	0.059 (2)	0.0036 (13)	−0.0135 (14)	−0.0211 (15)
C19	0.0340 (15)	0.0252 (13)	0.0260 (14)	0.0059 (11)	−0.0154 (12)	−0.0083 (11)
C20	0.0439 (17)	0.0275 (15)	0.0385 (16)	0.0023 (13)	−0.0162 (14)	−0.0076 (12)
C21	0.065 (2)	0.0254 (16)	0.053 (2)	−0.0028 (15)	−0.0207 (17)	−0.0041 (14)
C22	0.074 (2)	0.0238 (16)	0.058 (2)	0.0129 (16)	−0.0295 (19)	−0.0128 (15)
C23	0.055 (2)	0.0371 (17)	0.0500 (19)	0.0236 (15)	−0.0226 (16)	−0.0235 (15)
C24	0.0398 (16)	0.0338 (16)	0.0370 (16)	0.0084 (13)	−0.0143 (13)	−0.0146 (13)
C25	0.0248 (13)	0.0192 (13)	0.0256 (13)	0.0011 (10)	−0.0047 (11)	−0.0043 (10)
C26	0.0364 (16)	0.0211 (14)	0.0310 (15)	0.0033 (11)	−0.0011 (12)	−0.0029 (11)
C27	0.0353 (15)	0.0220 (13)	0.0266 (14)	0.0019 (11)	−0.0053 (12)	0.0013 (11)
C28	0.0237 (13)	0.0229 (13)	0.0225 (13)	−0.0025 (10)	−0.0034 (10)	−0.0026 (10)
C29	0.0234 (13)	0.0258 (13)	0.0205 (13)	−0.0048 (11)	−0.0029 (10)	−0.0019 (10)
C30	0.0233 (13)	0.0245 (13)	0.0201 (13)	−0.0014 (10)	−0.0036 (10)	−0.0041 (10)
C31	0.0312 (15)	0.0349 (15)	0.0172 (13)	0.0019 (12)	−0.0004 (11)	−0.0054 (11)
C32	0.0318 (15)	0.0301 (15)	0.0204 (13)	0.0049 (11)	−0.0019 (11)	−0.0078 (11)
C33	0.0225 (13)	0.0233 (13)	0.0221 (13)	0.0005 (10)	−0.0027 (10)	−0.0050 (10)
C34	0.0243 (13)	0.0245 (13)	0.0265 (14)	0.0010 (11)	−0.0032 (11)	−0.0081 (11)
C35	0.0233 (13)	0.0245 (13)	0.0223 (13)	0.0025 (11)	−0.0004 (10)	−0.0020 (10)
C36	0.0242 (13)	0.0257 (13)	0.0280 (14)	0.0011 (11)	−0.0036 (11)	−0.0040 (11)
C37	0.0236 (14)	0.0378 (16)	0.0238 (14)	0.0067 (12)	−0.0042 (11)	−0.0066 (12)
C38	0.0309 (15)	0.0411 (17)	0.0252 (14)	0.0032 (13)	−0.0024 (12)	0.0038 (12)
C39	0.0319 (15)	0.0376 (16)	0.0319 (16)	−0.0070 (13)	0.0004 (12)	0.0035 (13)
C40	0.0324 (15)	0.0317 (15)	0.0256 (14)	−0.0046 (12)	−0.0036 (12)	−0.0030 (11)
C41	0.0337 (15)	0.0244 (14)	0.0244 (14)	0.0016 (11)	0.0024 (12)	−0.0034 (11)
C42	0.0323 (17)	0.061 (2)	0.093 (3)	0.0073 (15)	−0.0122 (18)	−0.050 (2)
C43	0.045 (2)	0.054 (2)	0.104 (3)	−0.0023 (17)	0.001 (2)	−0.052 (2)
C44	0.055 (2)	0.0324 (17)	0.061 (2)	0.0126 (15)	0.0128 (18)	−0.0148 (16)
C45	0.069 (3)	0.102 (3)	0.077 (3)	0.057 (2)	−0.030 (2)	−0.046 (3)
C46	0.056 (2)	0.084 (3)	0.062 (2)	0.0368 (19)	−0.0269 (18)	−0.048 (2)
C47A	0.038 (2)	0.040 (2)	0.039 (2)	0.0023 (19)	−0.0091 (19)	0.0141 (19)
C47B	0.023 (5)	0.041 (6)	0.040 (6)	−0.009 (4)	−0.009 (4)	−0.004 (4)
C48A	0.063 (2)	0.038 (2)	0.108 (3)	−0.0171 (18)	0.006 (2)	−0.008 (2)
C48B	0.063 (2)	0.038 (2)	0.108 (3)	−0.0171 (18)	0.006 (2)	−0.008 (2)
C49A	0.036 (2)	0.042 (2)	0.035 (2)	−0.0070 (19)	−0.0023 (19)	−0.0026 (19)
C49B	0.032 (5)	0.039 (6)	0.038 (6)	−0.008 (4)	−0.020 (4)	0.011 (4)
C50A	0.0380 (19)	0.054 (2)	0.072 (2)	0.0066 (16)	0.0001 (17)	−0.0049 (19)
C50B	0.0380 (19)	0.054 (2)	0.072 (2)	0.0066 (16)	0.0001 (17)	−0.0049 (19)
C51A	0.034 (2)	0.051 (3)	0.063 (3)	0.001 (2)	−0.024 (2)	−0.017 (2)
C51B	0.023 (5)	0.029 (5)	0.030 (5)	−0.003 (4)	−0.010 (4)	0.010 (4)
C52A	0.057 (2)	0.0359 (18)	0.072 (2)	−0.0098 (16)	−0.0217 (18)	−0.0050 (17)
C52B	0.057 (2)	0.0359 (18)	0.072 (2)	−0.0098 (16)	−0.0217 (18)	−0.0050 (17)
C53A	0.030 (2)	0.035 (2)	0.041 (2)	−0.0074 (18)	−0.0176 (18)	0.0106 (18)
C53B	0.049 (6)	0.043 (6)	0.017 (5)	−0.001 (5)	−0.007 (4)	−0.001 (4)
C54A	0.046 (2)	0.062 (2)	0.099 (3)	0.0162 (18)	0.025 (2)	0.020 (2)
C54B	0.046 (2)	0.062 (2)	0.099 (3)	0.0162 (18)	0.025 (2)	0.020 (2)
Cl1A	0.094 (2)	0.141 (3)	0.116 (3)	0.010 (2)	−0.0330 (17)	−0.088 (2)
Cl2A	0.178 (4)	0.257 (8)	0.076 (3)	0.039 (5)	0.004 (2)	0.008 (4)
Cl1B	0.115 (6)	0.134 (9)	0.165 (11)	0.025 (6)	−0.063 (7)	−0.095 (8)
Cl2B	0.089 (5)	0.121 (7)	0.066 (5)	−0.013 (5)	−0.022 (3)	−0.037 (5)
Cl1C	0.345 (19)	0.085 (6)	0.247 (15)	−0.047 (8)	−0.204 (15)	0.035 (7)
Cl2C	0.230 (12)	0.140 (8)	0.073 (6)	−0.061 (8)	−0.033 (6)	−0.029 (5)

### Geometric parameters (Å, º)

**Table:**

Fe1—C1	1.982 (3)	C30—C31	1.435 (3)
Fe1—C1^i^	1.982 (3)	C31—C32	1.343 (3)
Fe1—N3^i^	1.9928 (18)	C31—H31	0.9500
Fe1—N3	1.9928 (18)	C32—C33	1.440 (3)
Fe1—N4	2.0038 (17)	C32—H32	0.9500
Fe1—N4^i^	2.0038 (17)	C33—C34	1.392 (3)
Fe2—C2	1.973 (2)	C34—C25^ii^	1.390 (3)
Fe2—C2^ii^	1.973 (2)	C34—C41	1.498 (3)
Fe2—N6^ii^	1.9945 (19)	C35—C40	1.393 (3)
Fe2—N6	1.9946 (19)	C35—C36	1.401 (3)
Fe2—N5^ii^	2.0056 (19)	C36—C37	1.387 (3)
Fe2—N5	2.0056 (19)	C36—H36	0.9500
N1—C1	1.156 (3)	C37—C38	1.375 (4)
N2—C2	1.155 (3)	C37—H37	0.9500
N3—C3	1.378 (3)	C38—C39	1.379 (4)
N3—C6	1.383 (3)	C38—H38	0.9500
N4—C11	1.377 (3)	C39—C40	1.382 (3)
N4—C8	1.380 (3)	C39—H39	0.9500
N5—C28	1.381 (3)	C40—H40	0.9500
N5—C25	1.385 (3)	C41—C46	1.359 (4)
N6—C33	1.378 (3)	C41—C42	1.378 (4)
N6—C30	1.382 (3)	C42—C43	1.393 (4)
N7—C47B	1.442 (9)	C42—H42	0.9500
N7—C51A	1.497 (4)	C43—C44	1.355 (5)
N7—C53B	1.500 (8)	C43—H43	0.9500
N7—C49A	1.514 (4)	C44—C45	1.343 (5)
N7—C47A	1.525 (4)	C44—H44	0.9500
N7—C53A	1.537 (4)	C45—C46	1.390 (4)
N7—C51B	1.562 (8)	C45—H45	0.9500
N7—C49B	1.567 (9)	C46—H46	0.9500
C3—C12^i^	1.397 (3)	C47A—C48A	1.571 (5)
C3—C4	1.437 (3)	C47A—H47A	0.9900
C4—C5	1.348 (3)	C47A—H47B	0.9900
C4—H4	0.9500	C47B—H47C	0.9900
C5—C6	1.433 (3)	C47B—H47D	0.9900
C5—H5	0.9500	C48A—H48A	0.9800
C6—C7	1.401 (3)	C48A—H48B	0.9800
C7—C8	1.385 (3)	C48A—H48C	0.9800
C7—C13	1.506 (3)	C49A—C50A	1.571 (5)
C8—C9	1.443 (3)	C49A—H49A	0.9900
C9—C10	1.348 (3)	C49A—H49B	0.9900
C9—H9	0.9500	C49B—H49C	0.9900
C10—C11	1.434 (3)	C49B—H49D	0.9900
C10—H10	0.9500	C50A—H50A	0.9800
C11—C12	1.392 (3)	C50A—H50B	0.9800
C12—C3^i^	1.397 (3)	C50A—H50C	0.9800
C12—C19	1.496 (3)	C51A—C52A	1.555 (5)
C13—C14	1.379 (4)	C51A—H51A	0.9900
C13—C18	1.390 (4)	C51A—H51B	0.9900
C14—C15	1.386 (4)	C51B—H51C	0.9900
C14—H14	0.9500	C51B—H51D	0.9900
C15—C16	1.373 (5)	C52A—H52A	0.9800
C15—H15	0.9500	C52A—H52B	0.9800
C16—C17	1.364 (5)	C52A—H52C	0.9800
C16—H16	0.9500	C53A—C54A	1.586 (5)
C17—C18	1.400 (4)	C53A—H53A	0.9900
C17—H17	0.9500	C53A—H53B	0.9900
C18—H18	0.9500	C53A—H54E	1.19 (3)
C19—C24	1.386 (4)	C53B—H53C	0.9900
C19—C20	1.397 (4)	C53B—H53D	0.9900
C20—C21	1.388 (4)	C54A—H54A	0.9800
C20—H20	0.9500	C54A—H54B	0.9800
C21—C22	1.376 (4)	C54A—H54C	0.9800
C21—H21	0.9500	C54A—H54D	0.9801 (10)
C22—C23	1.377 (4)	C54A—H54E	0.9801 (10)
C22—H22	0.9500	C54A—H54F	0.9801 (10)
C23—C24	1.395 (4)	C1A—Cl1A	1.733 (8)
C23—H23	0.9500	C1A—Cl2A	1.735 (9)
C24—H24	0.9500	C1A—H1A1	0.9900
C25—C34^ii^	1.390 (3)	C1A—H1A2	0.9900
C25—C26	1.438 (3)	C1B—Cl1B	1.662 (15)
C26—C27	1.342 (3)	C1B—Cl2B	1.730 (15)
C26—H26	0.9500	C1B—H1B1	0.9900
C27—C28	1.437 (3)	C1B—H1B2	0.9900
C27—H27	0.9500	C1C—Cl1C	1.697 (16)
C28—C29	1.393 (3)	C1C—Cl2C	1.720 (16)
C29—C30	1.396 (3)	C1C—H1C1	0.9900
C29—C35	1.497 (3)	C1C—H1C2	0.9900
			
C1—Fe1—C1^i^	180.00 (13)	N5—C25—C26	109.6 (2)
C1—Fe1—N3^i^	92.38 (8)	C34^ii^—C25—C26	124.3 (2)
C1^i^—Fe1—N3^i^	87.62 (8)	C27—C26—C25	107.4 (2)
C1—Fe1—N3	87.62 (8)	C27—C26—H26	126.3
C1^i^—Fe1—N3	92.38 (8)	C25—C26—H26	126.3
N3^i^—Fe1—N3	180.00 (10)	C26—C27—C28	107.5 (2)
C1—Fe1—N4	89.31 (8)	C26—C27—H27	126.2
C1^i^—Fe1—N4	90.69 (8)	C28—C27—H27	126.2
N3^i^—Fe1—N4	90.51 (7)	N5—C28—C29	125.7 (2)
N3—Fe1—N4	89.49 (7)	N5—C28—C27	109.7 (2)
C1—Fe1—N4^i^	90.69 (8)	C29—C28—C27	124.6 (2)
C1^i^—Fe1—N4^i^	89.31 (8)	C28—C29—C30	123.4 (2)
N3^i^—Fe1—N4^i^	89.49 (7)	C28—C29—C35	119.2 (2)
N3—Fe1—N4^i^	90.51 (7)	C30—C29—C35	117.3 (2)
N4—Fe1—N4^i^	180.00 (8)	N6—C30—C29	126.3 (2)
C2—Fe2—C2^ii^	180.0	N6—C30—C31	110.0 (2)
C2—Fe2—N6^ii^	90.31 (9)	C29—C30—C31	123.7 (2)
C2^ii^—Fe2—N6^ii^	89.69 (9)	C32—C31—C30	107.4 (2)
C2—Fe2—N6	89.69 (8)	C32—C31—H31	126.3
C2^ii^—Fe2—N6	90.31 (9)	C30—C31—H31	126.3
N6^ii^—Fe2—N6	180.0	C31—C32—C33	107.2 (2)
C2—Fe2—N5^ii^	90.82 (9)	C31—C32—H32	126.4
C2^ii^—Fe2—N5^ii^	89.18 (9)	C33—C32—H32	126.4
N6^ii^—Fe2—N5^ii^	90.06 (8)	N6—C33—C34	126.1 (2)
N6—Fe2—N5^ii^	89.94 (8)	N6—C33—C32	109.9 (2)
C2—Fe2—N5	89.18 (9)	C34—C33—C32	124.0 (2)
C2^ii^—Fe2—N5	90.82 (9)	C25^ii^—C34—C33	123.5 (2)
N6^ii^—Fe2—N5	89.94 (8)	C25^ii^—C34—C41	118.3 (2)
N6—Fe2—N5	90.06 (8)	C33—C34—C41	118.2 (2)
N5^ii^—Fe2—N5	180.00 (10)	C40—C35—C36	118.4 (2)
C3—N3—C6	105.30 (18)	C40—C35—C29	123.4 (2)
C3—N3—Fe1	126.50 (15)	C36—C35—C29	118.2 (2)
C6—N3—Fe1	127.82 (15)	C37—C36—C35	120.6 (2)
C11—N4—C8	105.63 (18)	C37—C36—H36	119.7
C11—N4—Fe1	126.77 (15)	C35—C36—H36	119.7
C8—N4—Fe1	127.59 (15)	C38—C37—C36	120.1 (2)
C28—N5—C25	105.70 (19)	C38—C37—H37	120.0
C28—N5—Fe2	127.35 (15)	C36—C37—H37	120.0
C25—N5—Fe2	126.91 (15)	C37—C38—C39	120.0 (2)
C33—N6—C30	105.54 (19)	C37—C38—H38	120.0
C33—N6—Fe2	127.45 (15)	C39—C38—H38	120.0
C30—N6—Fe2	127.00 (15)	C38—C39—C40	120.5 (2)
C47B—N7—C51A	171.9 (4)	C38—C39—H39	119.7
C47B—N7—C53B	116.8 (5)	C40—C39—H39	119.7
C51A—N7—C53B	63.8 (4)	C39—C40—C35	120.5 (2)
C47B—N7—C49A	68.0 (4)	C39—C40—H40	119.8
C51A—N7—C49A	112.2 (3)	C35—C40—H40	119.8
C53B—N7—C49A	173.4 (3)	C46—C41—C42	117.0 (3)
C47B—N7—C47A	79.6 (4)	C46—C41—C34	121.7 (2)
C51A—N7—C47A	107.4 (3)	C42—C41—C34	121.2 (2)
C53B—N7—C47A	66.7 (4)	C41—C42—C43	121.2 (3)
C49A—N7—C47A	110.9 (3)	C41—C42—H42	119.4
C47B—N7—C53A	62.1 (4)	C43—C42—H42	119.4
C51A—N7—C53A	110.9 (3)	C44—C43—C42	120.2 (3)
C53B—N7—C53A	80.2 (2)	C44—C43—H43	119.9
C49A—N7—C53A	106.4 (2)	C42—C43—H43	119.9
C47A—N7—C53A	109.1 (2)	C45—C44—C43	119.2 (3)
C47B—N7—C51B	106.6 (5)	C45—C44—H44	120.4
C51A—N7—C51B	66.5 (4)	C43—C44—H44	120.4
C53B—N7—C51B	109.6 (5)	C44—C45—C46	120.9 (3)
C49A—N7—C51B	72.1 (4)	C44—C45—H45	119.5
C47A—N7—C51B	173.8 (4)	C46—C45—H45	119.5
C53A—N7—C51B	74.6 (3)	C41—C46—C45	121.4 (3)
C47B—N7—C49B	112.3 (5)	C41—C46—H46	119.3
C51A—N7—C49B	74.7 (4)	C45—C46—H46	119.3
C53B—N7—C49B	103.7 (4)	N7—C47A—C48A	110.4 (3)
C49A—N7—C49B	69.8 (4)	N7—C47A—H47A	109.6
C47A—N7—C49B	69.3 (4)	C48A—C47A—H47A	109.6
C53A—N7—C49B	174.3 (4)	N7—C47A—H47B	109.6
C51B—N7—C49B	107.6 (5)	C48A—C47A—H47B	109.6
N1—C1—Fe1	177.2 (2)	H47A—C47A—H47B	108.1
N2—C2—Fe2	179.3 (2)	N7—C47B—H47C	111.4
N3—C3—C12^i^	126.2 (2)	N7—C47B—H47D	111.4
N3—C3—C4	110.14 (19)	H47C—C47B—H47D	109.2
C12^i^—C3—C4	123.7 (2)	N7—C49A—C50A	112.6 (3)
C5—C4—C3	107.2 (2)	N7—C49A—H49A	109.1
C5—C4—H4	126.4	C50A—C49A—H49A	109.1
C3—C4—H4	126.4	N7—C49A—H49B	109.1
C4—C5—C6	107.2 (2)	C50A—C49A—H49B	109.1
C4—C5—H5	126.4	H49A—C49A—H49B	107.8
C6—C5—H5	126.4	N7—C49B—H49C	109.8
N3—C6—C7	125.5 (2)	N7—C49B—H49D	109.8
N3—C6—C5	110.2 (2)	H49C—C49B—H49D	108.3
C7—C6—C5	124.0 (2)	N7—C51A—C52A	114.0 (3)
C8—C7—C6	123.6 (2)	N7—C51A—H51A	108.7
C8—C7—C13	119.3 (2)	C52A—C51A—H51A	108.7
C6—C7—C13	117.1 (2)	N7—C51A—H51B	108.7
N4—C8—C7	125.7 (2)	C52A—C51A—H51B	108.7
N4—C8—C9	109.9 (2)	H51A—C51A—H51B	107.6
C7—C8—C9	124.2 (2)	N7—C51B—H51C	109.1
C10—C9—C8	106.9 (2)	N7—C51B—H51D	109.1
C10—C9—H9	126.5	H51C—C51B—H51D	107.8
C8—C9—H9	126.5	N7—C53A—C54A	109.5 (3)
C9—C10—C11	107.4 (2)	N7—C53A—H53A	109.8
C9—C10—H10	126.3	C54A—C53A—H53A	109.8
C11—C10—H10	126.3	N7—C53A—H53B	109.8
N4—C11—C12	125.7 (2)	C54A—C53A—H53B	109.8
N4—C11—C10	110.2 (2)	H53A—C53A—H53B	108.2
C12—C11—C10	124.2 (2)	N7—C53A—H54E	136.5 (14)
C11—C12—C3^i^	123.7 (2)	C54A—C53A—H54E	38.09 (17)
C11—C12—C19	118.3 (2)	H53A—C53A—H54E	74.3
C3^i^—C12—C19	117.9 (2)	H53B—C53A—H54E	109.5
C14—C13—C18	118.5 (2)	N7—C53B—H53C	111.7
C14—C13—C7	120.8 (2)	N7—C53B—H53D	111.7
C18—C13—C7	120.6 (2)	H53C—C53B—H53D	109.5
C13—C14—C15	120.8 (3)	C53A—C54A—H54D	151 (2)
C13—C14—H14	119.6	H54A—C54A—H54D	52.8
C15—C14—H14	119.6	H54B—C54A—H54D	64.8
C16—C15—C14	120.2 (3)	H54C—C54A—H54D	99.0
C16—C15—H15	119.9	C53A—C54A—H54E	48.7 (15)
C14—C15—H15	119.9	H54A—C54A—H54E	106.6
C17—C16—C15	120.2 (3)	H54B—C54A—H54E	65.0
C17—C16—H16	119.9	H54C—C54A—H54E	142.9
C15—C16—H16	119.9	H54D—C54A—H54E	109.42 (16)
C16—C17—C18	120.0 (3)	C53A—C54A—H54F	98 (2)
C16—C17—H17	120.0	H54A—C54A—H54F	143.7
C18—C17—H17	120.0	H54B—C54A—H54F	82.4
C13—C18—C17	120.3 (3)	H54C—C54A—H54F	36.4
C13—C18—H18	119.8	H54D—C54A—H54F	109.41 (16)
C17—C18—H18	119.8	H54E—C54A—H54F	109.42 (16)
C24—C19—C20	118.4 (2)	Cl1A—C1A—Cl2A	117.7 (5)
C24—C19—C12	122.5 (2)	Cl1A—C1A—H1A1	107.9
C20—C19—C12	119.0 (2)	Cl2A—C1A—H1A1	107.9
C21—C20—C19	120.8 (3)	Cl1A—C1A—H1A2	107.9
C21—C20—H20	119.6	Cl2A—C1A—H1A2	107.9
C19—C20—H20	119.6	H1A1—C1A—H1A2	107.2
C22—C21—C20	120.3 (3)	Cl1B—C1B—Cl2B	110.6 (10)
C22—C21—H21	119.9	Cl1B—C1B—H1B1	109.5
C20—C21—H21	119.9	Cl2B—C1B—H1B1	109.5
C21—C22—C23	119.7 (3)	Cl1B—C1B—H1B2	109.5
C21—C22—H22	120.2	Cl2B—C1B—H1B2	109.5
C23—C22—H22	120.2	H1B1—C1B—H1B2	108.1
C22—C23—C24	120.4 (3)	Cl1C—C1C—Cl2C	112.0 (10)
C22—C23—H23	119.8	Cl1C—C1C—H1C1	109.2
C24—C23—H23	119.8	Cl2C—C1C—H1C1	109.2
C19—C24—C23	120.5 (3)	Cl1C—C1C—H1C2	109.2
C19—C24—H24	119.8	Cl2C—C1C—H1C2	109.2
C23—C24—H24	119.8	H1C1—C1C—H1C2	107.9
N5—C25—C34^ii^	126.1 (2)		

Symmetry codes: (i) −*x*, −*y*+1, −*z*; (ii) −*x*+1, −*y*, −*z*+1.
